# Monitoring the Effects of Hypolipidemic Treatment in Children with Familial Hypercholesterolemia in Poland

**DOI:** 10.3390/life10110270

**Published:** 2020-11-04

**Authors:** Matylda Hennig, Agnieszka Brandt-Varma, Anna Wołoszyn-Durkiewicz, Joanna Bautembach-Minkowska, Marta Buraczewska, Dominik Świętoń, Agnieszka Mickiewicz, Andrzej Rynkiewicz, Marcin Gruchała, Janusz Limon, Bartosz Wasąg, Magdalena Chmara, Mieczysław Walczak, Małgorzata Myśliwiec

**Affiliations:** 1 The Department of Paediatrics, Diabetology and Endocrinology, Faculty of Medicine, Medical University of Gdansk, 80-210 Gdansk, Poland; annanataliawoloszyn@gmail.com (A.W.-D.); bauti@wp.pl (J.B.-M.); malgorzata.mysliwiec@gumed.edu.pl (M.M.); 2 Surrey and Sussex Healthcare NHS Trust, Canada Avenue, Redhill RH15RH, UK; agnieszka.brandt@gumed.edu.pl; 3 The Department of Neonatology, Specialized Hospital in Wejherowo, 84-200 Wejherowo, Poland; szymanskam@gazeta.pl; 4 The Department of Radiology, Faculty of Medicine, Medical University of Gdansk, 80-210 Gdansk, Poland; smidon@gumed.edu.pl; 5 The Department of Cardiology I, Faculty of Medicine, Medical University of Gdansk, 80-210 Gdansk, Poland; amickiewicz@gumed.edu.pl (A.M.); marcin.gruchala@gumed.edu.pl (M.G.); 6 The Department of Cardiology and Cardiosurgery, Ist Cardiology Clinic, University of Warmia and Mazury in Olsztyn, 10-719 Olsztyn, Poland; andrzej.rynkiewicz@uwm.edu.pl; 7 The Department of Biology and Genetics, Faculty of Medicine, Medical University of Gdańsk, 80-210 Gdansk, Poland; jlimon@gumed.edu.pl (J.L.); bartosz.wasag@gumed.edu.pl (B.W.); magdalena.chmara@gumed.edu.pl (M.C.); 8 The Department of Paediatrics, Endocrinology, Diabetology, Metabolic Diseases and Developmental Cardiology, Faculty of Medicine, Pomeranian Medical University, 70-204 Szczecin, Poland; ghmwal@pum.edu.pl

**Keywords:** hypercholesterolemia, carotid intima-media thickness, cardiovascular disease, atherosclerosis

## Abstract

Familial hypercholesterolemia (FH) is the most common monogenic autosomal dominant disorder. FH results in an increased cardiovascular mortality rate. However, cardiovascular risk control factors enable the avoidance of approximately 80% of strokes and cardiovascular diseases. Therefore, early detection and implementation of lipid-lowering treatment is essential. In the present study, 57 pediatric patients aged 9.57 ± 3.26 years with FH were enrolled in the study. Researchers checked the lipid profile and performed the ultrasound imaging including intima-media thickness (IMT) measurement and echo (e)-tracking in the study group. Patients were treated with a low-cholesterol diet solely or along with pharmacological treatment with statins. Subsequently, patients were monitored for 12 months. The positive results of dietary treatment were observed in 40 patients. The efficacy of 12 months of nutritional therapy along with pharmacological treatment was reported in 27 patients. We observed a significant decrease in the carotid beta index stiffness and an insignificant decrease in the IMT in the group of patients treated with statins. The obtained data show that statin therapy in children with FH allow for the reduction of the degree of atherosclerotic vessel changes.

## 1. Introduction

Familial hypercholesterolemia (FH) is the most common monogenic autosomal dominant disorder caused by genetic alterations in the low-density lipoprotein receptor (LDLR), apolipoprotein B (APOB) or proprotein convertase subtilisin/kexin type 9 (PCSK9) genes. It is estimated that the cardiovascular (CV) mortality rate for a group of FH patients between the ages of 20 and 39 years is 100 times higher than in the general population [1]. However, approximately 80% of cardiovascular diseases (CVDs) and strokes may be avoided by risk control factors (World Health Organization—WHO Report, Gaining Health, 2006) [2]. Therefore, early disease detection and administration of appropriate lipid-lowering treatment are crucial. Homozygous FH (HoFH) is characterized by a more severe course of the disorder with symptoms appearing in early childhood. However, heterozygous FH (HeFH), asymptomatic in childhood, is diagnosed in the vast majority of patients [1,3]. Therefore, a family medical history of hypercholesterolemia and premature CV disease (before the ages 50–60 years) may be helpful in disease diagnosis, and this indicates the necessity of lipid profile assessment. In children with a (1) low-density lipoprotein cholesterol (LDL-C) level >190 mg/dL (4.9 mmol/L), (2) a family history and LDL-C >160 mg/dL (4.1 mmol/L), or (3) parents with FH and a LDL-C >130 mg/dL (3.4 mmol/L), FH can be diagnosed [4]. However, a definitive diagnosis can only be provided by molecular genetic testing [1].

High cholesterol levels in serum can damage the vessels, even without the coexistence of any other CV risk factors [5]. The effects of lipid-lowering treatment and noninvasive diagnostic tools evaluating the progression of atherosclerosis are still the subject of ongoing studies [6,7]. In clinical practice, several techniques are already being used such as intima-media thickness (IMT) measurement and echo (e)-tracking assessment of carotid artery stiffness by β-index evaluation [8,9].

The carotid artery is the usual site of IMT measurement due to that fact of its exceptional elasticity, low-resistance flow spectrum, and the small diameter of the muscular layer of the artery. Therefore, the increase of carotid IMT is considered as a development in atherosclerotic changes in arteries [8]. Researchers have reported positive correlations between IMT thickness and factors such as the older age of the patient, male gender, overweight, hypertension, hypercholesterolemia, hyperhomocysteinemia, elevated fibrinogen, and C-reactive protein (CRP) levels as well as carbohydrate metabolism disorders [10,11]. The measurement of carotid IMT was implemented as a primary prevention in patients without any clinical signs of CVD [11,12]. Moreover, the correlation between epithelium–tunica media thickness and organ complications, such as myocardial infarction (MI) or stroke, has also been found [13,14].

Echo-tracking is a noninvasive and repeatable examination that allows for the assessment of arterial stiffness. Changes in arterial structure may indicate the presence of atherosclerosis long before the clinical manifestation of the disease [15]. Parameters evaluated in echo-tracking, such as stiffness index β, Young’s modulus of elasticity, index of blood vessels compliance, pulse–wave velocity, and augmentation index, are correlated with the age of the patient, hypertension, and hypercholesterolemia [8,16]. E-tracking is believed to be more precise and repeatable than IMT measurement. In addition, e-tracking detects atherosclerotic changes in blood vessels earlier than IMT examination [14,17].

The early use of statins in patients with FH can keep LDL-C levels similar to healthy individuals [18]. Nevertheless, researchers have not intensively studied the possibility of reversing the atherosclerotic process or preventing progression of atherosclerosis via the administration of proper clinical management such as dietary treatment, intensification of physical activities, and introduction of lipid-lowering therapy with statins in children. Therefore, this study aimed to monitor the effects of lipid-lowering therapy in children with FH to analyze the impact of early diagnosis and proper treatment implementation on the clinical course and atherosclerotic process.

## 2. Materials and Methods

Fifty-seven pediatric patients (girls: 33; boys: 24, F:M ratio: 1.4) aged 9.57 ± 3.26 years (standard deviation: 3.88; interquartile range: 9.67) with a diagnosis of FH, confirmed by molecular testing, were enrolled in the study. At the moment of admission to the Lipid Outpatient Clinic, the youngest patient was 1.5 years old and the oldest was 17.2 years old. Patients were divided into 4 age groups: Group 1: <6 years, Group 2: 6–10 years, Group 3: 10–15 years, Group 4: >15 years.

In all patients, serum total cholesterol, HDL-C, and triglyceride (TG) levels were tested. Laboratory tests were performed at the Central Laboratory of University Clinical Centre of Gdansk, Poland. Total cholesterol, HDL-C, and triglycerides were measured on Alinity analyzers (Abbot, Germany), using the enzyme-linked immunoassay method. Concentrations of the LDL-C fraction was calculated using the Friedewald formula, provided that the triglycerides level was under 400 mg/dl. Secondary causes of lipid disorders, such as coeliac disease, hypothyroidism, and carbohydrates metabolism disorders, were ruled out in a studied group. Patients with documented lipid disorders were furtherly examined for atherosclerosis in carotid arteries with the use of ultrasound imaging including IMT measurement and e-tracking.

Patients received recommendations for a specific diet based on a small amount of animal fats and a high intake of vegetables, fruits, and olive oil. In addition, individuals were asked to increase fiber consumption and avoid products such as eggs, offal, fatty meat, and cheese. Finally, the intake of oils rich in monounsaturated fatty acids (oleic acid: olive oil, rape oil) and polyunsaturated fatty acids n-6 (linoleic acid: corn oil, thistle oil, soya oil, sunflower oil) was recommended.

Patients were treated with a low-cholesterol diet solely or along with pharmacological treatment with statins.

In order to monitor the efficacy of therapy, lipid profiles were reevaluated after six months of dietary treatment and after one year of pharmacological therapy. The positive results of the dietary treatment were observed in 40 patients.

### Imaging Parameters

In the studied group, the intima-media complex thickness (IMT) was examined with the use of carotid ultrasound. Patients were examined after 5 min of rest in the supine position, with the head in the axis of the body, turned slightly to the opposite direction of the examined site. IMT measurement was carried out via an Aloka Alpha 6 ultrasound machine (Hitachi, Tokyo, Japan, 2011) with the use of an 8 MHz linear transducer. Focal lengths were constant and image enhancement was selected as a way to achieve the smallest number of artefacts in the vessels’ lumen as possible. The transducer was placed perpendicular to the common carotid artery. The measurement was performed 2 cm from the left and right carotid sinus, and then the arithmetic mean from the received values was calculated. The results of mean intima-media complex thickness were referred to Jourdan and colleagues’ norms [9].

In addition, e-tracking was performed. The measurements were implemented before, during, and after dietary or pharmacological therapy. Patients were examined after 5 min rest, in the supine position with their head in the axis of the body, turned slightly to the opposite direction of the examined site. The examination was performed via an Aloka Alpha 6 ultrasound machine (Hitachi, Tokyo, Japan 2011) with the use of an 11 MHz linear transducer and automatic sphygmomanometer Omron M2 (Omron, Kyoto, Japan 2012). The blood pressure cuff was adjusted to the arm circumference of the patient. Blood pressure was measured on the arm artery. The transducer was placed perpendicular to the common carotid artery. The measurement was performed 2 cm from the left and right carotid sinus. and then the arithmetic mean from the received values was calculated. The examination concluded with the evaluation of the stiffness ß-index—the ratio of the natural logarithm of blood pressure changes to the changes in vessels’ diameter, arterial compliance (AC), augmentation index (AI), Young’s modulus of elasticity (Eo), and pulse-wave velocity (PWVß). The stiffness ß-index was elevated in case of increased vessel stiffness. The results of e-tracking were referenced to by Calabro and colleagues’ norms [8].

One specialist performed all of the imaging examinations at the Department of Radiology, University Clinical Centre in Gdansk, Poland.

## 3. Statistical Analysis

Collected data were analyzed statistically to verify the postulated hypothesis. ANOVA variation analysis was conducted to verify the hypothesis of equal means of particular trials. The non-parametric Kruskal–Wallis test was applied for heterogeneous variation groups and small groups of subjects (homogeneity of variance was assessed by the Bartlett’s test).

Paired student’s *t*-tests were carried out to test the equality of the mean parameters in dependent samples (before and after treatment) for the normal distribution group. The non-parametric Wilcoxon matched pairs test was applied for far from normal distribution groups (the normality of distribution was assessed by the Shapiro–Wilk test).

Correlation analysis was conducted by calculating Pearson’s correlation coefficient (r) for particular pairs of parameters. A *p*-value < 0.05 was claimed as statistically significant. Statistical analysis was carried out with the use of EPI INFO Ver. 7.1.1.14 (release date 2 July 2013) statistical software.

The study was approved by the Independent Bioethics Committee for Scientific Research at the Medical University in Gdansk, Poland (No 04/0136/09/156).

## 4. Results

### 4.1. The Characterization of Lipid Disorders in the Study Group

**Figure 1:**
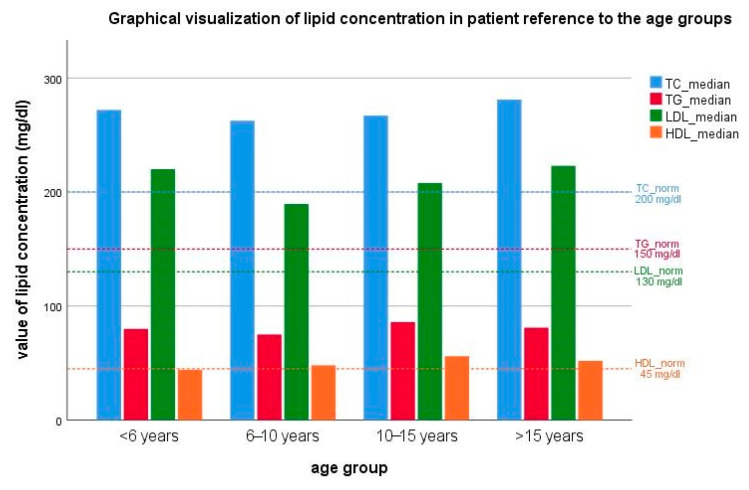
Graphical visualization of the median value of lipid concentration in patient reference to the age groups with the reference values (horizontal lines).

In the study group (*n* = 57), the mean total cholesterol (TC) concentration at the moment of admission to the Outpatient Clinic was 287 ± 67 mg/dL (7.42 ± 1.7 mmol/L), mean LDL-C concentration was 213 ± 73 mg/dL (5.5 ± 1.9 mmol/L), mean HDL-C concentration was 55.9 ± 26.5 mg/dL (1.4 ± 0.7 mmol/L), and mean TG concentration was 94.8 ± 48 mg/dL (1 ± 1.2 mmol/L). Statistical analysis did not reveal any significant correlations (*p*-value < 0.05) between the age of the patients and TC or cholesterol fraction concentrations. The highest deviation from TC and LDL-C reference norms in patients under 6 years old were observed, whereas the lowest deviation was reported in children between 6 and 10 years old. In addition, in this group of patients, the lowest TGs concentration was also noticed. Observations in particular groups did not show any significant differences in statistical analyses (Figure 1).

### 4.2. Vascular System Assessment in the Study Group

Ultrasonographic assessment of carotid IMT was carried out in 51 patients, whereas beta stiffness index was evaluated in 40 patients (e-tracking examination) at the beginning of dietary or pharmacological treatment. Carotid IMT and e-tracking examinations were not carried out in 6 and 17 patients, respectively, due to the lack of cooperation.

**Figure 2:**
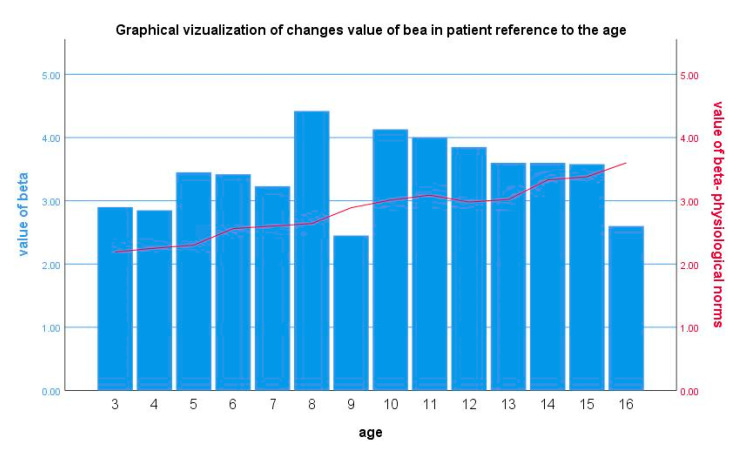
The analysis of carotid beta stiffness index changes in reference to physiological age-specific norms (*n* = 40).

**Figure 3:**
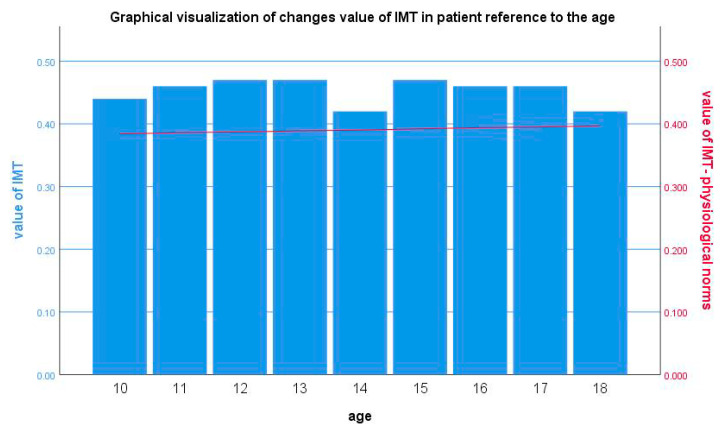
The analysis of carotid intima-media thickness (IMT) changes in reference to physiological norms (*n* = 51).

After one year of observation, researchers evaluated the efficacy of the therapy with the use of IMT and e-tracking. IMT was performed in 32 patients (12 patients on a low-cholesterol diet and 20 patients who received pharmacological treatment), whereas e-tracking was carried out in 26 patients (11 patients on a low-cholesterol diet and 15 patients who received pharmacological treatment). Part of the study group did not report to the Radiology Department; thus, the radiologist was not able to conduct e-tracking for all patients due to the lack of cooperation. In all investigated individuals, changes in vascular system were observed (Figure 2 and Figure 3).

### 4.3. Multi-Factor Analysis of Vascular Changes

The nature of vascular changes in the e-tracking examination referred to the age, sex, nutrition of the patient, and to the lipid parameters. In the study, a statistically significant positive correlation between carotid beta stiffness index and the age of the patient was reported (Table 1, *p* = 0.046). On the other hand, an insignificant positive correlation was noted between arterial compliance (AC) and LDL-C concentration (Table 1, *p* = 0/073).

In further statistical analyses, an insignificant positive correlation between augmentation index (AI) and the age of the patient was revealed (Table 1, *p* = 0.078). Moreover, a significant positive correlation was found between the Young’s modulus of elasticity (Eo) as well as the one-point pulse wave velocity and body mass index (Table 1, *p* = 0.005).

According to a multivariate analysis based on multiple backward regression, the age of the patient was reported to be the factor influencing the beta stiffness index. In addition, body mass index was an independent factor affecting the local one-point pulse wave velocity (Table 1). To summarize, these data show that younger patients are characterized by lower arterial stiffness.

### 4.4. Assessment of Treatment Outcomes in Patients with FH

The comparative analysis of total, LDL, HDL-C, and TG concentrations was performed in a group of 40 patients at the beginning of treatment and after 6 months of a low-cholesterol diet. Seventeen patients did not report to the Laboratory Centre.

**Figure 4:**
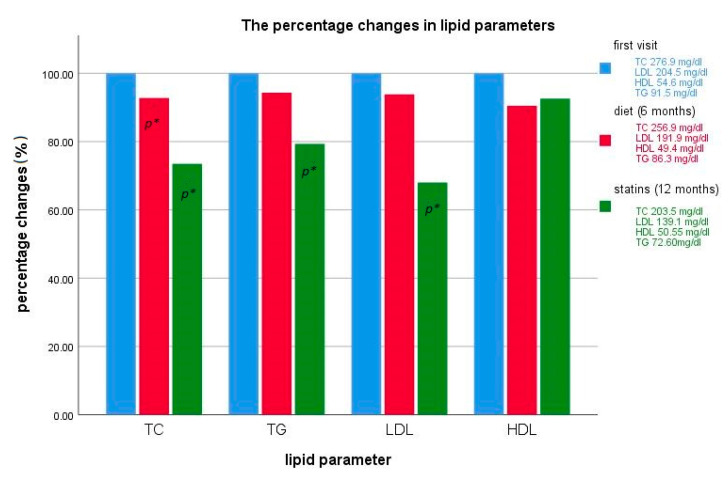
The percentage changes in total cholesterol, low-density lipoprotein cholesterol (LDL-C), high-density lipoprotein cholesterol (HDL-C), and triglyceride concentrations, depending on the method of treatment (i.e., low-cholesterol diet exclusively or simultaneously with statin therapy). Where p* means significant (*p* < 0.05) changes between first visit and reference treatment in values of lipid parameters.

The implementation of the diet resulted in a decrease in mean TC levels by 20 mg/dL (0.5 mmol/L) (7.2%), a decrease in LDL-C levels of 12.6 mg/dL (0.3 mmol/L) (6.2%) as well as reduction in the HDL-C mean value concentration of 5.2 mg/dL (0.1 mmol/L) (9.5%) and a reduction in the concentration of triglycerides by 5.2 mg/dL (0.6 mmol/L) (5.7%) (Figure 4). Only the difference in TC levels, before and after dietary treatment, were reported as statistically significant (*p* < 0.001). Despite the decrease in total and LDL-C concentrations, its levels were still much higher than norms for their age (Table 2).

The efficacy of 12 months of nutrition therapy along with pharmacological treatment was reported in 27 patients (67.5%)

At a further stage of the study, a comparative assessment of particular lipid parameters was carried out in patients with FH during dietary and statin therapy, one year after implementation of the treatment. Twenty-seven patients out of 40 (67.5%) received statin therapy. The rosuvastatin doses ranged from 5 mg to 40 mg, the average of 12.63 ± 7.321 mg.

Mean TC level decreased by 85.9 mg/dL (2.22 mmol/L) (29.6%), mean LDL-C by 73.08 mg/dL (1.98 mmol/L) (34.4%), mean HDL-C level by 7.65 mg/dL (0.2 mmol/L) (13.3%), and triglycerides concentration by 28.6 mg/dL (0.32 mmol/L) (28.2%). Lipid parameters changes, before and after 12 months of statin therapy, were statistically significant for total cholesterol (*p* < 0.001), LDL-C (*p* < 0.001), triglycerides (*p* = 0.0015), and statistically non-significant for HDL-C (*p* = 0.46) (Figure 4).

Mean total and LDL-C concentrations were still above the upper limit of the normal range (3.5 mg/dL (0.09 mmol/L) and 9.1 mg/dL (0.24 mmol/L), respectively) (Table 2).

In the course of pharmacological treatment, apart from the decrease in total cholesterol levels, triglycerides and HDL-C concentrations were also reduced (Figure 4).

### 4.5. The Effects of Treatment on Vascular Changes

At the end of the observation, researchers assessed the relationship between vascular changes and the method of treatment (i.e., a low-cholesterol diet exclusively or simultaneously with statin therapy).

The analysis was carried out in two phases: preliminary examination and examination after one year of hypolipidemic diet, with or without statins. The initial analysis was performed during the course of the dietary treatment exclusively.

### 4.6. Vascular Changes Under the Influence of a Low-Cholesterol Diet

**Figure 5:**
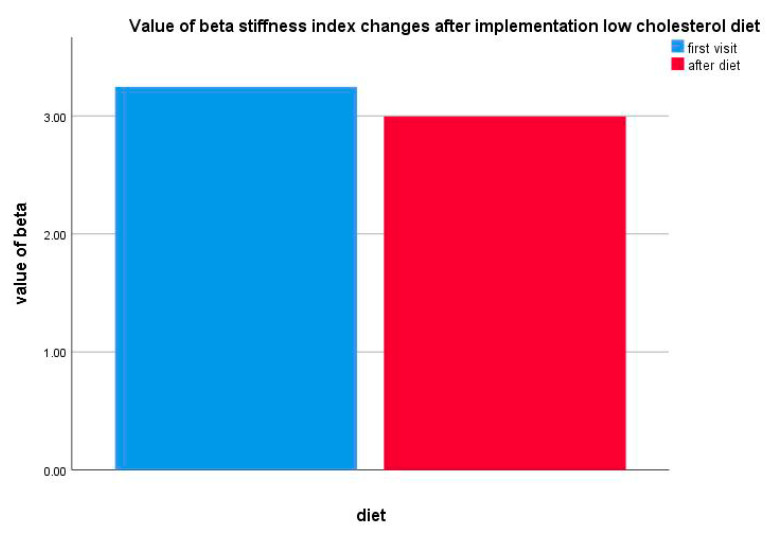
Value of beta stiffness index changes after a low-cholesterol diet implementation.

In the group of patients (*n* = 11) exclusively on the low-cholesterol diet, a decrease in the carotid beta index stiffness was observed; however, the changes were not statistically significant (*p* = 0.374) (Figure 5).

**Figure 6:**
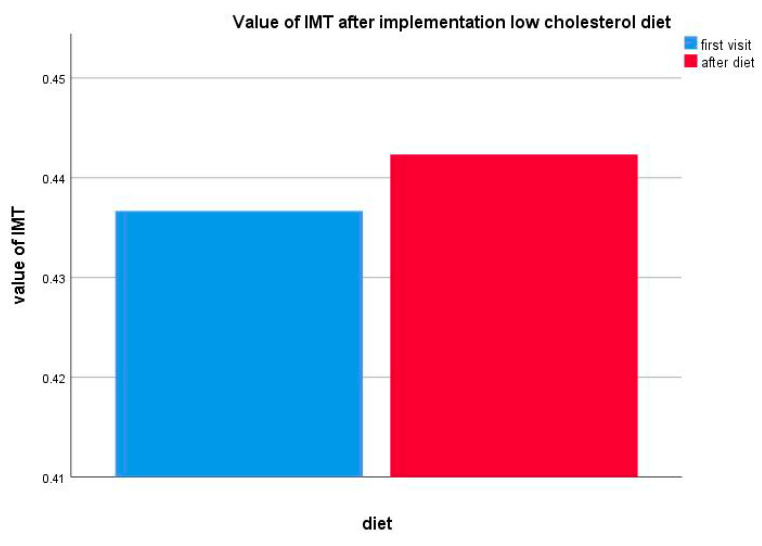
Value of IMT changes after a low-cholesterol diet implementation.

Moreover, a statistically significant difference was not reported for IMT changes in the group of patients (*n* = 12) on the low-cholesterol diet only (*p* = 0.594) (Figure 6).

### 4.7. Vascular Changes Under the Influence of Statin Therapy

**Figure 7:**
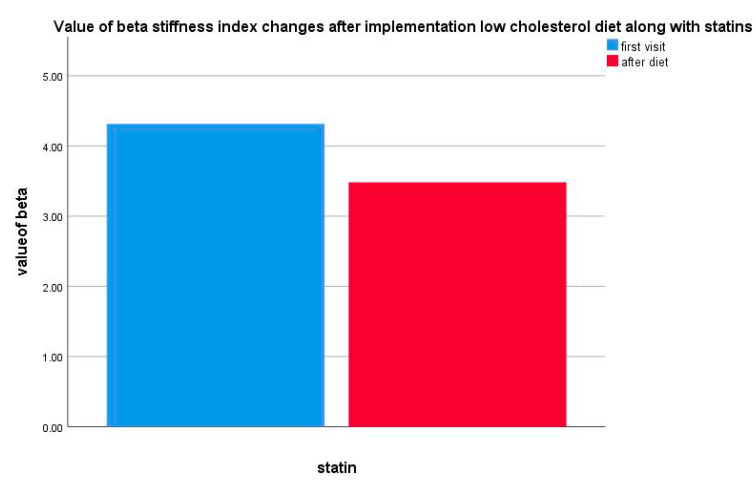
Value of beta stiffness index changes after implementation of a low-cholesterol diet along with statins.

In the group of patients (*n* = 15) on the pharmacological treatment, a significant decrease in the carotid beta index stiffness was reported (*p* = 0.031) (Figure 7).

**Figure 8:**
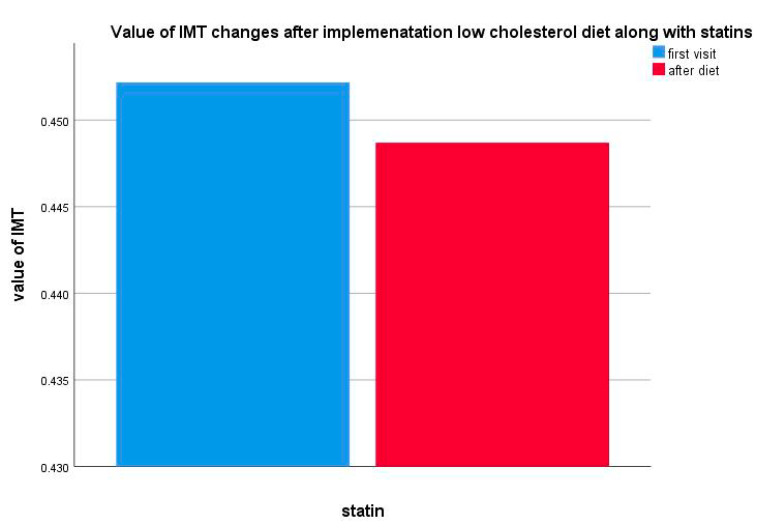
Value of IMT changes after implementation of a low-cholesterol diet along with statin therapy.

In the study group (*n* = 20), the IMT decrease was statistically insignificant during pharmacotherapy (*p* = 0.936) (Figure 8).

## 5. Discussion

In the majority of well-developed countries, cardiovascular diseases are the leading cause of death. The most common cause of cardiovascular diseases is atherosclerosis, which continues to develop from early childhood in patients with lipid disorders [19].

IMT measurement is a diagnostic technique for cardiovascular risk assessment in adults. In the pediatric population, certain analyses were performed to identify risk groups among patients with hypertension, diabetes, renal insufficiency, obesity, dyslipidemia, and homocysteinemia [9]. Jourdan and colleagues conducted an ultrasonography examination of 250 healthy children and adults. Based on the study, standard values of IMT in relation to age and height were established. Moreover, the study showed that IMT in healthy children increased with age, BMI index, and systolic pressure [9]. In contrast, Calabro et al. were unable to report a correlation between IMT and age in a group of 130 healthy children [8].

IMT in children with FH who are >6 years old was significantly higher at baseline compared with unaffected siblings in as study by Braamskamp and colleagues [20].

Based on Jourdan et al.’s criteria, vascular system abnormalities were detected in all individuals enrolled in their study. All patients were characterized, inter alia, by an increase in IMT. However, an insignificant decrease in IMT was reported in children aged 6–10 years old. Although IMT changed with age, the values were not statistically significant, similar to Calabro and colleagues [8,9].

E-tracking is another non-invasive imaging technique that allows for the assessment of the elasticity of arteries. This examination enables the evaluation of changes in artery structures— characteristics of atherosclerosis—long before clinical presentation of the disorder. Previous studies have indicated the positive correlation between parameters evaluated in e-tracking (e.g., stiffness β-index, Young’s modulus of elasticity, index of blood vessel compliance, pulse–wave velocity, augmentation index) and the age of the patient as well as the presence of hypertension or hypercholesterolemia. Based on Calabro et al.’s study conducted on a group of 130 healthy children, age-specific norms were set for this method [8].

Riggio et al. suggested that the e-tracking technique may allow for early detection of atherosclerotic changes and suggested that e-tracking parameters positively correlate with total cholesterol and LDL-C levels [16]. In accordance with these data, an increase in vascular stiffness with age until the patient was 15 years old was observed in the current study.

The slight decrease in vascular stiffness in the age group 15 to 18 years is puzzling. For this reason, the beta stiffness index in patients at the age of 16 was found to be within the age-specific normal range. The coexistence of another factor in this group of patients, such as an increase of sex hormones in serum, could be possible.

### The Effects of Hypolipidemic Therapy

Based on the previous studies, it is accepted that nutritional management allows for the reduction of cholesterol levels by 10–15% [20]. In the current study, cholesterol levels were analyzed in a group of 40 patients before and after 6 months on a hypolipidemic diet. The average total cholesterol level decreased by 7.2%, which was statistically significant. The reduction of the LDL-C level by 6.2% was also noted. The decrease in cholesterol concentration was significantly lower than in previously published data. The reduction of HDL-C was unexpected. It possibly indicates that more attention should be paid to patients’ education regarding proper nutrition on lipid disorders. Despite the decrease of total and LDL-C levels in serum, its concentrations were still above the normal range. We can conclude that the low-cholesterol diet was not sufficient in children with FH as well as in adults. Therefore, pharmacotherapy implementation at the early stage of treatment is necessary.

The early introduction of pharmacological treatment seems to be efficient in the decrease in cardiovascular events in patients with FH [21]. Luirink et al. have proved that the implementation of statin therapy in childhood has a protective role against the development of cardiovascular complications. Moreover, researchers have observed a slower progression of carotid IMT in patients who started treatment with statins in childhood [22]. Statins may reduce LDL-C concentration by 10–60%, depending on the dosage [21,23]. In the current study, the results of statin therapy were analyzed every year after treatment implementation. After one year of therapy, total and LDL-C levels were still above the normal range. However, statins turned out to be much more effective than the low-cholesterol diet.

The effects of dietary and statin therapy were observed in ultrasonography imaging as well. Wiegman et al. reported a significant IMT decrease in the group of 106 pediatric patients treated with pravastatin for 24 months, whereas the increase in IMT was noted in the control group of 108 patients [23]. The decrease in LDL-C concentrations and IMT reductions during treatment were statistically significant. In the Braamskamp study, children under Rosuvastatin treatment for 2 years resulted in a slower progression of carotid IMT [20]. Kusters et al., in a ten-year follow-up, showed that patients treated with statins had a similar increase in IMT thickness as healthy children [21].

In our study, patients on pharmacological treatment were followed-up after one year. In the group of children treated exclusively with the low-cholesterol diet, IMT increases were reported, whereas the group treated with statins achieved IMT decreases. However, observed changes were statistically insignificant, and the results were in accordance with previously published reports.

Further studies are required to explain the impact of lipid-lowering pharmacological treatment on vascular changes in children with FH. Researchers should thoroughly investigate the effect of novel therapies, such as proprotein convertase subtilisin–kexin type 9 inhibitors (PCSK-9). Only a few studies have focused on the effects of PCSK-9 inhibitors in the pediatric population. Santos et al. have shown that evolocumab, a monoclonal antibody against PCKS-9, reduced LDL-C levels by 77.5 mg/dL in children with FH after 24 weeks of treatment. Furthermore, Mandraffino et al. have proved that the addition of PCSK-9 inhibitors and ezetimibe treatment to the high-intensity statin therapy in adults allowed the reduction of the pulse–wave velocity along with improvements in LDL-C levels [24,25].

Riggio et al. showed that e-tracking not only allows to evaluate early atherosclerotic changes but also detects vascular changes in a more sensitive way than IMT measurement [16]. Moreover, researchers distinguished patients with FH and patients with hypercholesterolemia caused by other factors in the group with lipid disorders. This study has demonstrated that patients differ not only by total and LDL-C concentrations (patients with FH were characterized by significantly higher cholesterol levels) but also by vascular stiffness. Statistically significant differences between the group of patients with FH and individuals with hypercholesterolemia caused by other factors were not reported in IMT measurements. In accordance with previously published reports, not only cholesterol reduction but also a statistically significant decrease in vascular stiffness after one year of statin therapy were observed in the studied group. The decrease in vascular stiffness was also noted under the influence of diet, though the correlation was not statistically significant [16].

There is scarce evidence in the literature concerning the impact of statins on vascular stiffness changes in patients with hypercholesterolemia. Based on the current results, we assumed that e-tracking is a more useful tool for the evaluation of atherosclerotic changes in development. Non-invasiveness, repeatability, and detection of atherosclerotic changes at the early stage of the disease are undeniable advantages of this examination. By contrast, IMT measurement, which allows revealing atherosclerotic changes in vessels as well, is less sensitive and repeatable than e-tracking. Moreover, vascular changes are observed after longer exposure to adverse factors.

## 6. Conclusions

(1) Beta index stiffness evaluation in e-tracking imaging technology is more accurate when assessing the results of hypolipidemic treatment in pediatric FH patients than IMT measurement;
(2) Statin therapy in children with FH allows reducing the degree of atherosclerotic vessel changes;
(3) A low-cholesterol diet without statins is not sufficient to achieve a clinical effect.

## Figures and Tables

**Table 1:** Multivariate correlation in E-tracking Imaging Technology. Where “*p* =…*”—statistical significant; “+”—positive correlation; “−“—negative correlation, HDL-C—high-density lipoprotein cholesterol, LDL-C—low-density lipoprotein cholesterol).

	ß (N = 40)	AC (N = 37)	AI% (N = 40)	PWVß (N = 37)	Ep (N = 37)
**Age**	**+** (*p* = 0.046 *)	**−** (*p* = 0.089)	**+** (*p* = 0.078)		
**HDL-C**		**−** (*p* = 0.083)			
**LDL-C**		**+** (*p* = 0.073)			
**BMI**				**+** (*p* = 0.005 *)	**+** (*p* = 0.005 *)

**Table 2:** Lipid parameters before and after 12 months of treatment with statins (*n* = 27).

	Total Cholesterol [mg/dL]	LDL [mg/dL]	HDL [mg/dL]	Triglicerydes [mg/dL]
Diet	289.40 ± 59.84	212.20 ± 71.16	58.22 ± 31.90	101.20 ± 64.30
12 months statins	203.50 ± 34.80	139.10 ± 32.10	50.55 ± 9.65	72.60 ± 33.37
*p*-value	<0.001	<0.001	0.456	0.002
